# Alcoholic drink produced by pea is a risk factor for incident knee surgery in patients with knee osteoarthritis

**DOI:** 10.3389/fnut.2023.1264338

**Published:** 2023-10-17

**Authors:** Xiaopeng Huang, Jinshan Zhang, Yongqiang Zheng, Xiaofeng Liu, Yongquan Xu, Yangzhen Fang, Zhenyu Lin, Liang Lin, Hongpeng Zhang, Zefeng Wang

**Affiliations:** Orthopedics Department, Jinjiang Municipal Hospital, Fujian, China

**Keywords:** knee osteoarthritis, alcoholic drink, Kellgren-Lawrence (K-L) grades, total knee arthroplasty (TKA), unicompartmental knee arthroplasty (UKA), high tibial osteotomy

## Abstract

**Objective:**

The objective of this study is to investigate whether alcohol exposure and specific alcoholic drinks are independent risk factors for incident knee surgery in knee osteoarthritis (KOA) patients.

**Methods:**

We identified all patients who were clinically diagnosed as KOA between January 2010 and January 2018 in our outpatient department. Demographic, clinical, and radiographic data were collected from the database of our hospital. Next, we analyzed the association between alcohol consumption and incident knee surgery.

**Results:**

A total of 4,341 KOA patients completed the current study and were included in the final analysis. Incident knee surgery for the purpose of treating osteoarthritis was observed in 242 patients. Incident knee surgery was significantly associated with age (OR [95%CI], 1.023 [1.009–1.039], *P* = 0.002), BMI (OR [95%CI], 1.086 [1.049–1.123], *P* < 0.001), baseline K-L grade 3 (OR [95%CI], 1.960 [1.331–2.886], *P* = 0.001), baseline K-L grade 4 (OR [95%CI], 1.966 [1.230–3.143], *P* = 0.005), 7.1–14 drinks per week (OR [95%CI], 2.013 [1.282–3.159], *P* = 0.002), >14 standard drinks per week (OR [95%CI], 2.556 [1.504–4.344], *P* = 0.001), and the most common alcoholic drink produced by pea (OR [95%CI], 3.133 [1.715–5.723], *P* < 0.001).

**Conclusion:**

KOA patients who consumed more than seven standard drinks per week were at substantial risk of incident knee surgery. In addition, alcoholic drink produced by pea is also an independent risk factor.

## 1. Introduction

Knee osteoarthritis (KOA) is characterized by three core symptoms (pain, stiffness, and limited function) and accompanied by many structural alterations including degradation of cartilage subchondral bone remodeling, meniscal degeneration, and Hoffa's and effusion synovitis, affecting more than 10% of the overall population globally as estimated (1–3). The disease burden of KOA had been projected to double in the following decades because of the increasing aging of the population (4). Many risk factors for KOA development and progression including female sex, aging, and overweight/obesity have been well-established by previous studies (5, 6). Lifestyle intervention is the cornerstone of KOA management. According to a previous study, weight loss could be beneficial for KOA patients in the long term (7). In addition, increasing physical activities appropriately is also important for KOA patients by increasing lower-limb muscle strength (8–10).

KOA could also be treated with surgical procedures. The efficacy and safety of total knee arthroplasty (TKA), unicompartmental knee arthroplasty (UKA), and high tibial osteotomy (HTO) have been well-established and generally recommended for KOA management (11, 12). In contrast, many high-quality, multicenter, randomized clinical trials have consistently and repeatedly demonstrated that arthroscopic procedures, including lavage, debridement, and arthroscopic partial meniscectomy, are ineffective and even harmful in the long term for KOA patients (13–16). However, this high-quality evidence failed to curb the increase in arthroscopic procedures in KOA patients (13–16). Nevertheless, incident arthroscopic procedures at least reflected poor symptom control and were reasonably considered clinically important events for disease progression.

Excessive alcohol consumption and alcoholism are major global risk factors for increased all-cause mortality and incident morbidities but not limited to cardiovascular diseases, malignancies, neurological diseases, and accidental injuries (17–20). For KOA, a previous study revealed that excessive alcohol drinking was associated with an increased risk of KOA (21). Furthermore, the mechanistic link between alcohol intake and KOA development has been elucidated by a preclinical study (22). A population-based study concluded that alcohol consumption contributed to radiographic change in KOA in Korea (23). Notably, previous studies in KOA patients only focused on the amount of alcohol consumption rather than specific types of alcoholic drinks. This study aimed to investigate whether alcohol exposure and specific alcoholic drinks are independent risk factors for incident knee surgery in KOA patients.

## 2. Patients and methods

### 2.1. Study population

**Figure 1:**
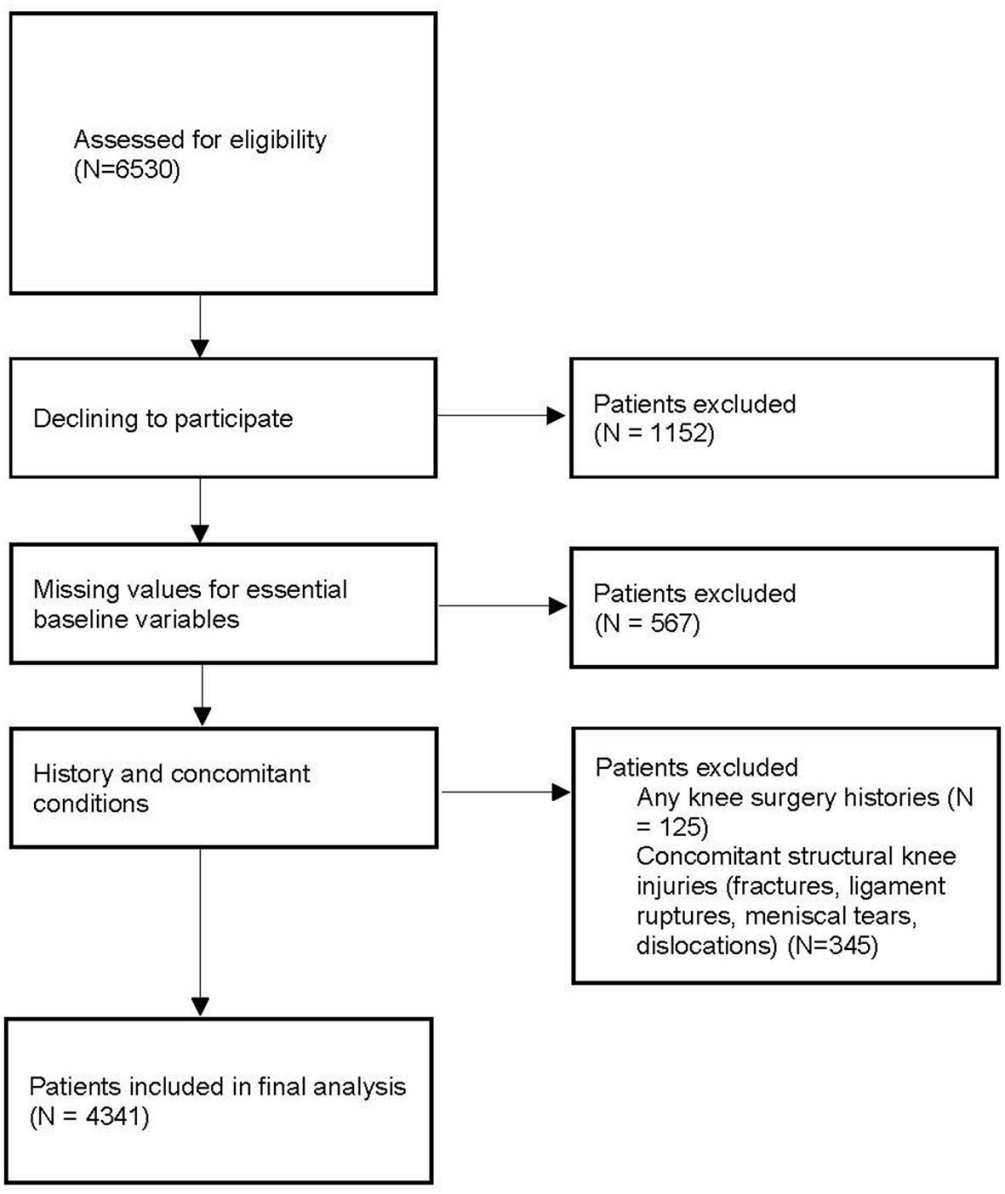
Study screening and enrollment.

This study followed the Declaration of Helsinki and all local laws and regulations during design and conducted data analysis. We obtained ethics approval for collecting all related data from patients and medical records. We identified all patients who were clinically diagnosed as KOA at a visit to our outpatient department between January 2010 and January 2018 via the hospital information system (HIS). The clinical diagnosis of KOA in the current study was defined as those made by clinical specialists in orthopedic and/or sports medicine. It was generally determined based on patient history, physical examination, and laboratory and radiographic findings (24). As shown in Figure 1, patients were excluded from this study if they had any knee surgery histories (*n* = 125), concomitant structural knee injuries (fractures, ligament ruptures, meniscal tears, and dislocations; *n* = 345), missing values for essential baseline variables (*n* = 567), and declining to participate (*n* = 1,152).

### 2.2. Baseline patient data

We defined the baseline as the time of performing the first knee plain radiograph during the study period. All baseline demographic and clinical data were retrieved from HIS, and the information was further confirmed by contacting patients through on-site interview, telephone, email, and instant message software. The baseline demographic data consisted of demographic information (sex, age, and body mass index [BMI]) calculated by weight, height, and education. The Kellgren–Lawrence (K-L) grades (25) were rated by a radiographic evaluation committee consisting of three radiologists specialized in musculoskeletal radiology. The rating process was conducted without grouping information. The consensus on grading was achieved by the majority of people. When the two knees had different K-L grades, the final K-L grade was recorded according to the more severe side.

### 2.3. Alcohol consumption and details of alcoholic drinks

The alcohol consumption was self-reported by patients based on their recalling for the last 12 months. Show cards were used to prompt recalling of the number of standard drinks usually consumed per week. Each show card, respectively, illustrated the typical volume of Chinese distilled spirit (25 ml of 50% alcohol/volume), Chinese rice wine (90 ml of 15% alcohol/volume), wine (120 ml of 11% alcohol/volume), and beer (285 ml of 4.5% alcohol/volume) equivalent to 10 g of ethanol, defined as a standard drink (26). For those who reported frequent consumption of other types of alcoholic beverages during the previous year, researchers calculated the weekly consumption after collecting the label information of these alcoholic beverages. The weekly number of standard drinks was recorded in a categorical manner as none, ≤1, 1.1–7, 7.1–14, and >14 standard drinks per week. The researchers also asked the patients for his or her most common alcoholic beverage type (beer, Chinese distilled spirit, Chinese rice wine, wine, and others). We collected label information of the most commonly consumed alcoholic beverages and recorded the raw materials (barley, wheat, grape, rice, kaoliang, pea, and corn) of these alcoholic beverages.

### 2.4. Definition of incident knee surgery

In this study, incident knee surgery was defined as any surgical procedure performed for the purpose of treating KOA no matter whether this type of surgical procedure was recommended or not. The incident knee surgery and types of surgery were reported by patients. In the current study, incident knee surgery included TKA, arthroscopic procedures (ineffective and not recommended), UKA, and HTO.

### 2.5. Statistical analysis

All statistical analyses were performed using SPSS software (IBM Corp. Released 2019. IBM SPSS Statistics for Windows, version 26.0. Armonk, NY: IBM Corp). The statistical significance was set at a two-sided 0.05. We first tabulated descriptive statistics to summarize the characteristics of the subjects. Continuous and categorical variables were, respectively, presented as means ± standard deviations and counts (percentage), unless otherwise indicated. When the *P*-value was <0.2 in univariable analysis, the variables along with demographic variables (age, sex, and BMI) were further included in logistic regression for multivariable analysis.

## 3. Results

A total of 4,341 KOA patients were included in the final analysis. Incident knee surgery for the purpose of treating osteoarthritis was observed in 242 patients out of 4,341 patients during the study period. Specifically, 65 patients had TKA, 5 had UKA, 162 had arthroscopic procedures, and 10 had high tibial osteotomy. For univariable analyses, incident knee surgery was significantly associated with age, BMI, baseline K-L grades, alcohol consumption, and the most common type of alcoholic beverage produced by pea (Table 1). The logistic regression model included sex, baseline BMI, baseline age, alcohol consumption, most common type of alcoholic beverage produced by pea, and baseline K-L grades.

**Table 1:** Univariable analysis on characteristics grouped by incident knee surgery.

	**No incident knee surgery (*n* = 4,099)**	**Incident knee surgery (*n* = 242)**	***P-*value**
Age, years	61.02 ± 8.83	62.93 ± 8.46	< 0.001
**Sex, No. (%)**			
Male	1,068 (26.1%)	70 (28.9%)	0.324
Female	3,031 (73.9%)	170 (71.1%)	
Baseline BMI, kg/m^2^	25.07 ± 3.59	26.26 ± 4.39	< 0.001
**Alcohol consumption, No. (%)**			
None	775 (18.9%)	29 (12.0%)	< 0.001
≤ 1 standard drink/week	784 (19.1%)	29 (12.0%)	
1.1–7 standard drinks/week	1,146 (28.0%)	68 (28.1%)	
7.1–14 standard drinks/week	1,008 (24.6%)	79 (32.6%)	
>14 standard drinks/week	386 (9.4%)	37 (15.3%)	
**Most common type of alcoholic beverage** ^*^ **, No. (%)**			
Beer	1,003 (30.2%)	59 (27.7%)	0.204
Chinese rice wine	318 (9.6%)	16 (7.5%)	
Wine	675 (20.3%)	37 (17.4%)	
Chinese distilled spirit	981 (29.5%)	81 (38.0%)	
Others	180 (5.4%)	10 (4.7%)	
Multiple	167 (5.0%)	10 (4.7%)	
**Most common type of alcoholic beverage produced by kaoliang, No. (%)**			
Yes	1,012 (24.7%)	86 (35.5%)	0.233
No	3,087 (75.3%)	156 (64.5%)	
**Most common type of alcoholic beverage produced by rice, No. (%)**			
Yes	1,068 (26.1%)	70 (28.9%)	0.324
No	3,031 (73.9%)	172 (71.1%)	
**Most common type of alcoholic beverage produced by barley, No. (%)**			
Yes	1,300 (31.7%)	81 (33.8%)	0.569
No	2,799 (68.3%)	161 (66.2%)	
**Most common type of alcoholic beverage produced by wheat, No. (%)**			
Yes	551 (13.4%)	39 (16.1%)	0.238
No	3,548 (86.6%)	203 (83.9%)	
**Most common type of alcoholic beverage produced by pea, No. (%)**			
Yes	79 (1.9%)	14 (5.8%)	< 0.001
No	4,020 (98.1%)	228 (94.2%)	
**Most common type of alcoholic beverage produced by grape, No. (%)**			
Yes	406 (9.9%)	22 (9.1%)	0.680
No	3,693 (90.1%)	220 (90.9%)	
**Most common type of alcoholic beverage produced by corn, No. (%)**			
Yes	205 (5.0%)	16 (6.6%)	0.268
No	3,894 (95.0%)	226 (93.4%)	
**Hypertension, No. (%)**			
Yes	1,335 (32.6%)	86 (35.5%)	0.339
No	2,764 (67.4%)	156 (64.5%)	
**K-L grades, No. (%)**			
0 or 1	1,134 (27.7%)	48 (19.8%)	0.001
2	1,766 (43.1%)	96 (39.7%)	
3	833 (20.3%)	66 (27.3%)	
4	366 (8.9%)	32 (13.2%)	
**Diabetes, No. (%)**			
Yes	505 (12.3%)	32 (13.2%)	0.678
No	3,594 (87.7%)	210 (86.8%)	
**Smoking, No. (%)**			
Yes	357 (8.7%)	24 (9.9%)	0.519
No	3,742 (91.3%)	218 (90.1%)	
**Education, No. (%)**			
More than 9 years	1,036 (25.3%)	66 (27.3%)	0.488
Not more than 9 years	3,063 (74.7%)	176 (72.7%)	

Data are shown as means (±SD) unless otherwise indicated.

BMI, body mass index; K-L, Kellgren–Lawrence.

^*^For drinkers only.

After adjustment with the multivariable logistic regression, incident knee surgery was significantly associated age (OR [95%CI], 1.023 [1.009–1.039], *P* = 0.002), BMI (OR [95%CI], 1.086 [1.049–1.123], *P* < 0.001), baseline K-L grade 3 (OR [95%CI], 1.960 [1.331–2.886], *P* = 0.001), baseline K-L grade 4 (OR [95%CI], 1.966 [1.230–3.143], *P* = 0.005), 7.1–14 drinks per week (OR [95%CI], 2.013 [1.282–3.159], *P* = 0.002), >14 standard drinks per week (OR [95%CI], 2.556 [1.504–4.344], *P* = 0.001), and the most common alcoholic drink produced by pea (OR [95%CI], 3.133 [1.715–5.723], *P* < 0.001; Table 2).

**Table 2:** Multivariable analysis on characteristics grouped by incident knee surgery.

	**Odds ratio (95% confidence interval)**	***P*-value**
Baseline BMI	1.086 (1.049–1.123)	< 0.001
Male Sex (Reference: female)	0.918 (0.676–1.247)	0.585
Age	1.023 (1.009–1.039)	0.002
Most common type of alcoholic beverage produced by pea	3.133 (1.715–5.723)	< 0.001
Alcohol consumption ≤ 1 standard drink/week (Reference: none)	0.939 (0.551–1.603)	0.819
Alcohol consumption 1.1–7 standard drinks/week (Reference: none)	1.536 (0.976–2.417)	0.064
Alcohol consumption 7.1–14 standard drinks/week (Reference: none)	2.013 (1.282–3.159)	0.002
Alcohol consumption >14 standard drinks/week (Reference: none)	2.556 (1.504–4.344)	0.001
K-L grade 2 (Reference: K-L grade 0 or 1)	1.339 (0.935–1.919)	0.111
K-L grade 3 (Reference: K-L grade 0 or 1)	1.960 (1.331–2.886)	0.001
K-L grade 4 (Reference: K-L grade 0 or 1)	1.966 (1.230–3.143)	0.005

The logistic regression model included baseline sex, baseline BMI, age, alcohol consumption, most common type of alcoholic beverage produced by pea, and K-L grades.

## 4. Discussion

A previous study using data from the Osteoarthritis Initiative study revealed that excessive alcohol drinking was associated with an increased risk of both radiographic and symptomatic KOA (21). Similarly, a population-based and longitudinal study conducted in Korea found that alcohol consumption contributed to the radiographic progression of KOA (23). For osteoarthritis in other anatomic sites, researchers reported that alcohol exposure is associated with structural destruction and inflammatory features of hand osteoarthritis (27). Notably, previous studies in KOA patients only focused on the amount of alcohol consumption rather than specific types of alcoholic drinks.

The underlying mechanism between alcohol drinking and osteoarthritis remains greatly unclear, while many plausible hypotheses and theories have been proposed. As revealed by many preclinical investigations, alcohol intake is capable of inducing pro-inflammatory states in joints and is thus believed to be a contributing factor to the development and progression of KOA (22). In a mouse model, chronic alcohol consumption also increases cartilage loss in large joints by impairing extracellular matrix production and accelerating the degradation (28). In addition, alcohol could increase the level of inflammatory mediator interleukin-6 (IL-6), an important cytokine in KOA development and progression (29).

The most important and novel finding of the current study is the unexpected association between incident knee surgery and exposure to pea-derived alcoholic beverages in KOA patients. To the best of our knowledge, only some types of Chinese distilled spirit (most of them are made by a fermentation technique called “Daqu”) use pea as a major raw material worldwide. Daqu is one of the oldest and most widely used fermentation technique for spirit-making (30). In addition to alcohol, fermentation with the Daqu technique often produces substantial amount and various types of chemicals with unknown effects on humans (30–32). Clearly, microbiota (molds, yeasts, and bacteria) are responsible for the final chemicals. However, in the current study, we are unable to further determine whether certain microorganisms are involved in this phenomenon. Nevertheless, our finding provides a unique and exciting insight into the pathogenesis of osteoarthritis.

The current study had several limitations. First, future confirmation of our observation by prospective and larger cohort studies should be performed. If so, mechanistic studies are urgently needed to explore why pea-derived alcoholic beverages are associated with osteoarthritis progression. Notably, this is the first study reporting this phenomenon, and thus, we are currently unable to propose a reasonable hypothesis without future mechanistic studies. Second, because of the observational nature, the decision on whether to receive surgical treatment in this study lacked transparency for us and readers. Finally, because the drinking pattern and specific types of alcoholic beverages may largely vary by age, sex, and socioeconomic status in a general population (33, 34), future studies on alcohol consumption and KOA should further explore these factors. Extrapolation of our conclusion to a different setting should be cautious.

## Data availability statement

The original contributions presented in the study are included in the article/supplementary material, further inquiries can be directed to the corresponding author.

## Ethics statement

The studies involving humans were approved by the Ethics Committee at Jinjiang Municipal Hospital. The studies were conducted in accordance with the local legislation and institutional requirements. The participants provided their written informed consent to participate in this study.

## Author contributions

XH: Data curation, Investigation, Writing—original draft. JZ: Conceptualization, Funding acquisition, Methodology, Project administration, Supervision, Validation, Writing—review and editing. YZ: Data curation, Investigation, Writing—original draft. XL: Data curation, Investigation, Writing—original draft. YX: Data curation, Investigation, Writing—original draft. YF: Data curation, Investigation, Writing—original draft. ZL: Data curation, Investigation, Writing—original draft. LL: Data curation, Investigation, Writing—original draft. HZ: Data curation, Investigation, Writing—original draft. ZW: Data curation, Investigation, Writing—original draft.
